# Associations among Stroke, Myocardial Infarction, and Amaurosis Fugax in a Tertiary Referral Hospital in Taiwan

**DOI:** 10.3390/jcm11175088

**Published:** 2022-08-30

**Authors:** Ming-Hui Sun, Nomin-Erdene Ognoo

**Affiliations:** 1 Department of Ophthalmology, Linkou Chang Gung Memorial Hospital, Taoyuan 333, Taiwan; 2 College of Medicine, Chang Gung University, Taoyuan 333, Taiwan; 3 Sondra Eye Clinic, Department of Ophthalmology, Mongolian National University of Medical Science, Ulaanbaatar 14210, Mongolia

**Keywords:** fugax amaurosis, carotid stenosis

## Abstract

Background: To determine the associations among carotid stenosis, stroke, and myocardial infarction (MI) in patients with amaurosis fugax (AF). Methods: We retrospectively reviewed the records of patients diagnosed as having AF between January 2000 and December 2019. Among 14,857 patients with AF initially reviewed in the database, only 173 were ultimately enrolled, after excluding patients with wrong diagnosis, insufficient medical records, or loss of follow-up. Results: Of the 173 patients with AF, 61 (35.3%) had carotid stenosis, and among them, 18 (10.4%) had severe stenosis. In multivariate regression analysis, carotid stenosis was significantly associated with age (*p* = 0.009), male sex (*p* = 0.006), and ischemic heart disease (*p* = 0.039). Sixteen (9.2%) patients experienced a stroke after AF diagnosis (mean time to stroke: 23.1 ± 31.1 months, range: 1 day~85 month), 11 (68.8%) of whom had carotid artery stenosis (*p* = 0.003). Three (1.7%) patients had MI after AF (mean time to MI: 24.8 ± 35.9 months, range: 12 days~66 months), none of whom had carotid artery stenosis (*p* = 0.553). Four (2.3%) patients had central retinal artery occlusion (CRAO) after AF, all of whom had carotid artery stenosis (*p* = 0.034). Conclusions: A high incidence of internal carotid artery stenosis was observed after AF attack and was significantly associated with stroke. The incidence of MI and CRAO after AF was low. Among them, only CRAO was associated with carotid artery stenosis.

## 1. Introduction

Amaurosis fugax (AF) is the sudden, temporary, partial, or total loss of vision from any cause [1]. Vision loss typically lasts from a few seconds to several minutes before a return to normal [1]. AF usually occurs in patients over the age of 50 years who have other vascular risk factors, including hypertension, hypercholesterolemia, heart disease, or previous episodes of transient ischemic attack (TIA) [2]. AF is considered a “vascular” cause of various types of transient visual loss, including retinal ischemia, optic nerve ischemia, and retinal migraine, and is an important symptom of occlusion or stenosis of the internal carotid artery circulation [3]. The prevalence of carotid artery stenosis in the general population is 4.1% [4]. In a retrospective study of 302 patients in Sweden, 18.9% of patients diagnosed as having AF had significant carotid artery stenosis [5]. McCullough et al. also noted that 20% of patients with AF were found to have a stenosis of 50% or greater in the ipsilateral internal carotid artery, as detected by duplex ultrasound scanning [6].

Stroke remains a major public health burden worldwide, with an estimated 16 million new stroke cases per year and a prevalence of over 60 million. In one study investigating stroke caused by large-vessel mechanisms, a stenosis of 50% or greater in extracranial internal carotid artery and a stenosis of 50% or greater in intracranial internal carotid artery were associated with 8.0% and 0.9% of ischemic stroke, respectively [7]. A prospective study revealed that cardiac embolism occurred in 8.7% of patients presenting with AF. Less commonly, cardiac emboli can originate from atrial fibrillation, valvular disease, atrial myxoma, mitral valve prolapse, and other sources within the heart, including mobile masses [8,9]. Cardiac disease, especially myocardial infarction or failure, occurs in 8% of patients presenting with AF [10]. The postulation that cardiogenic cerebral embolism is a frequent cause of stroke has gained increased prominence in recent years.

Patients with AF are at a high risk of stroke, cerebral TIA, retinal (central or branch) artery occlusion, adverse cardiac diseases, and death. Therefore, in this study, we aimed to determine the association between stroke and myocardial infarction in patients with AF.

## 2. Material and Methods

**Figure 1:**
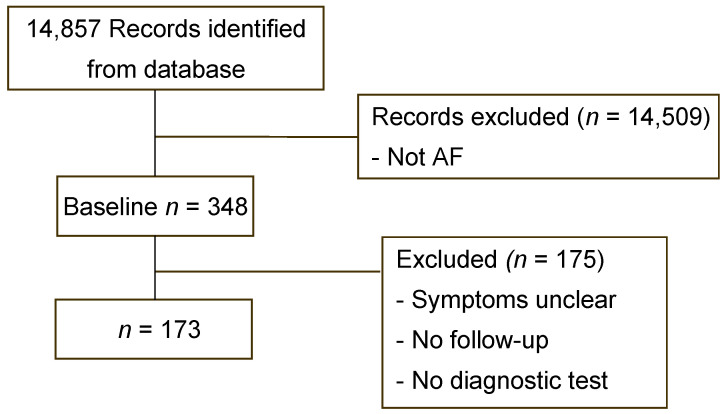
Inclusion and exclusion criteria for patients with AF. AF: amaurosis fugax.

This retrospective comparative cohort study was conducted at Chang Gung Memorial Hospital (CGMH), Taoyuan, Taiwan. We retrospectively reviewed the medical records of patients diagnosed as having AF according to the International Classification of Diseases, 9th Revision (362.34) or 10th Revision (G45.3) and who attended Linkou Chang Gung Memorial Hospital between January 2000 and December 2019. According to this classification, 14,857 patients with ICD-9 coding for 362.34 or ICD-10 coding for G45.3 were selected. After a review of their medical records, it was determined that 14,509 patients were not strictly AF cases, as they did not have typical presentation or symptoms; this left 348 patients who had diagnoses of AF. Among these patients, 175 were excluded due to having unclear visual symptoms, unsatisfactory imaging diagnostic tests, or no follow-up examinations. Ultimately, we enrolled 173 patients in this study (Figure 1).

Our patients’ data included age, sex, medical history of systemic and ocular disorders, smoking history, and specification of monocular or binocular symptoms during AF episodes. The medical specialties of clinicians with whom patients had been in contact for AF treatment and the results of the diagnostic imaging tests were also recorded. All patients with AF were subjected to transcranial Doppler (TCD), transcranial color Doppler (TCCD), computed tomography angiography (CTA) of brain, magnetic resonance angiography (MRA) of the brain, or carotid angiography. Categories of stenosis were defined as follows: 0–20% of the carotid lumen occluded was considered mild stenosis, 21% to 50% of the carotid lumen occluded was classified as moderate stenosis, 51% to 70% of the carotid lumen occluded was considered moderate–severe stenosis, and 71% to 99% of the carotid lumen occluded was defined as severe carotid stenosis.

## 3. Statistics

All statistical analyses were performed using SPSS version 23.0 (IBM Corp, Armonk, NY, USA). A *p* value less than 0.05 was considered statistically significant.

## 4. Results

A total of 348 patients met the initial eligibility criteria, and thereafter, another 175 patients were excluded. The mean follow-up time was 45.6 ± 44.8 months. Patient demographics and clinical characteristics are presented in Table 1.

**Figure 2:**
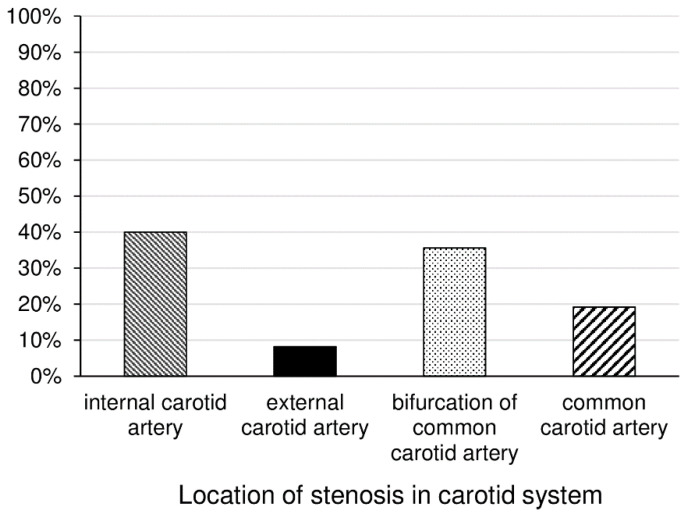
Location of stenosis in carotid system.

In all, 173 patients met all inclusion criteria and were enrolled. Among them, 85 (49.1%) patients were women and 88 (50.9%) were men. Regarding carotid artery stenosis prevalence, 112 (64.7%) patients did not have carotid artery stenosis and 61 (35.3%) patients did. Three (1.7%), 33 (19.1%), 7 (4%), and 18 (10.4%) patients had mild, moderate, moderate–severe, and severe carotid artery stenosis, respectively. Among those with carotid artery stenosis, 44 (72.1%) patients were male and 17 (27.9%) patients were female (*p* < 0.001). In terms of mean age, patients with carotid artery stenosis were significantly older than those without carotid stenosis (61.4 ± 15.1 vs. 59.0 ± 16.2 years, respectively; *p* = 0.002). Regarding the location of stenosis in the carotid system, internal carotid artery (40%) and bifurcation of common carotid artery (35.6%) were the most common sites, followed by common carotid artery (19.2%) and external carotid artery (8.2%) (Figure 2).

Smoking (*p* = 0.004), hypertension (*p* = 0.046), coronary artery disease (*p* = 0.004), ischemic heart disease (*p* = 0.014), and prior stroke (*p* = 0.003) were significantly associated with carotid artery stenosis. Interestingly, patients with migraine were less associated with carotid stenosis (*p* = 0.046). Hyperlipidemia’s association with carotid artery stenosis was almost significant (*p* = 0.067), and diabetes mellitus (*p* = 0.176) was not significantly associated with carotid artery stenosis. In the multivariate analysis, factors significantly associated with carotid artery stenosis were age (odds ratio [OR]: 1.03, 95% CI, 1.00–1.06, *p* = 0.009), male sex (OR:2.84, 95% CI, 1.84–5.99, *p* = 0.006), and ischemic heart disease (OR:2.56, 95% CI, 1.05–6.25, *p* = 0.039).

Of the patients, four developed central retinal artery occlusion (CRAO), and one patient developed central retinal vein occlusion (CRVO) after AF in our study. Among those, four (80%) patients (all patients with CRAO) had carotid artery stenosis. CRAO was significantly associated with carotid artery stenosis (*p* = 0.034). Among those CRAO patients with carotid artery stenosis, two patients had moderate carotid artery stenosis, one had severe carotid artery stenosis, and one had total occlusion. Of those patients with CRAO, three (75%) had a stroke. Our study found that CRAO was significantly associated with stroke (*p* < 0.001). Three patients (1.7%) were diagnosed as having nonarteritic anterior ischemic optic neuropathy (NAION), but such a diagnosis was not significantly (*p* = 0.251) associated with carotid artery stenosis.

Among our patients, 71 (41%) visited an ophthalmologist for their first AF-related medical consultation. In terms of presentation symptoms, 23 (13.3%) patients had binocular AF symptoms, and 150 (86.7%) patients had monocular AF symptoms. Regarding the number of attack episodes, 69 (39.9%) patients had had a single episode of transient visual loss, and 104 (60.1%) patients had had multiple episodes. Of the 157 patients who remembered the duration of their AF episodes, 117 (74.5%) patients recalled visual symptoms lasting for seconds to within 10 min; only 40 (25.5%) patients’ visual symptoms persisted for 10 min or longer, and they then recovered totally. Nevertheless, laterality (unilateral or bilateral), number of episodes, and duration of AF symptoms were not significantly associated with carotid artery stenosis (*p* = 0.677, 0.449, and 0.666, respectively) (Table 2).

**Figure 3:**
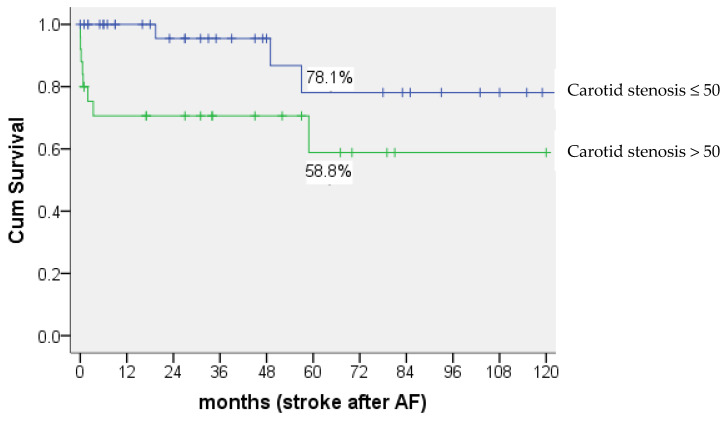
Kaplan-Meier curves showing the survival probability. AF: amaurosis fugax.

Of the 173 patients, 16 (9.2%) experienced a stroke after AF (mean time to stroke: 23.1 ± 31.1 months, range: 1 day~85 month). Among those patients, 11 (92.7%) had carotid artery stenosis. Carotid artery stenosis was significantly associated with stroke (*p* = 0.003). Furthermore, the percentage of development of stroke after AF was 29.4% (5 of 17) in patients with carotid stenosis >70%, 37.5% (3 of 8) in patients with carotid stenosis between 51%–70%, 9.1% (3 of 33) in patients with carotid stenosis between 21%–50%, and 0% (none of 3) in patients with carotid stenosis <20%, respectively. There was a significant difference in survival time between patients with carotid stenosis >50% and patients with carotid stenosis ≤50%. By 5 years after AF, 78.1% of those with carotid stenosis ≤50% had no stroke, compared with 58.8% of those with carotid stenosis >50% (*p* = 0.024) (Figure 3).

The average time to develop stroke after AF was 131.1 ± 12.5 months in patients with carotid stenosis ≤50%, compared with 77.8 ± 12.2 months in patients with carotid stenosis >50%.

Myocardial infarction (MI) was observed in three (1.7%) patients overall (mean time to MI: 24.8 ± 35.9 months, range: 12 days~66 months), and none of these patients had carotid artery stenosis (*p* = 0.553).

Of the 173 patients who presented with AF, carotid angiography or stenting was carried out in 52.9% of (nine of seventeen) patients with carotid stenosis >70%, compared with 37.5% of (three of eight) patients with stenosis between 51%–70%, 6.1% of (two of thirty-three) patients with stenosis between 21%–50%, and 0% of (none of three) patients with stenosis <20, showing that the main indication for angioplasty or stenting was carotid stenosis >50% in this study. However, some patients with carotid stenosis <50% also underwent angioplasty or stenting, which suggests these patients might have had other vascular risk factors. Overall, 14 (8.1%) patients underwent carotid angioplasty or stenting, and 53 (30.6%) patients received antithrombotic therapy alone.

## 5. Discussion

In our study, 29.5% of patients with AF underwent TCD or TCCD only for detecting carotid stenosis, while the others underwent both ultrasound and CTA, MRA, or carotid angiography if the ultrasound findings were inconclusive. Although carotid angiography has the highest sensitivity, prior study has shown that there is good agreement between Doppler and CTA, MRA, or carotid angiography in determining carotid artery stenosis [11].

The overall prevalence of carotid stenosis was 35.3%, and severe carotid stenosis (≥70%) had a prevalence of 10.4% among participants of our study, which was lower than that observed in the study of Kvickstrom et al. (18.9%) [5]. The percentage of patients who had experienced a stroke after AF was 9.2% in our study. Our results on the prevalence of carotid artery stenosis in patients with stroke are consistent with those in the work of Flaherty et al. [7]. Our study also revealed that stroke was associated with carotid artery stenosis. The stroke rate has been reported to be approximately 1.5% to 1.8% in prior studies [10,12]. Zhang et al. evaluated the risk of concurrent acute ischemic stroke and monocular vision loss of vascular etiology through brain MRI, reporting that 19.5% of patients had positive findings for acute cortical stroke on brain MRI [13].

Among those with carotid artery stenosis, 41.0% of patients had stenosis >50% (stenosis 51%–70%: 13.1%; stenosis >70%: 27.9%) in our study. In accordance with the North American Symptomatic Carotid Endarterectomy Trial, stenosis <70% of the luminal diameter was classified as moderate carotid stenosis and 70% to 99% of occlusion was classified as severe stenosis [14]. According to that study, severe carotid stenosis (70% or more) was associated with higher risk of stroke relative to moderate carotid stenosis (30%–69%), and the risk of stroke could be reduced by 17% if patients with severe carotid stenosis received carotid endarterectomy. However, the gain from surgery was smaller in patients with moderate carotid stenosis. Therefore, those with carotid stenosis over 70% or more were considered to have high risk of stroke, and needed more attention. In this study, percentage of stroke after AF in patients with carotid stenosis >70% was a little lower than in patients with carotid stenosis between 51%–70% (29.4% vs. 37.5%). We think that this might be related to the higher rate of angioplasty or stenting undertaken in patients with carotid stenosis >70% than in those with carotid stenosis between 51%–70% (52.9% vs. 37.5%). Overall, our study showed carotid stenosis > 50% was associated with higher risk of stroke compared to carotid stenosis ≤50% within 5 years. As in previous studies, male sex, age, smoking, hypertension, prior stroke, carotid artery disease, and ischemic heart disease were identified as risk factors for carotid artery stenosis [2,5].

In this study, four patients developed central retinal artery occlusion (CRAO), and one patient developed central retinal vein occlusion (CRVO) after AF. Considering the definition of AF, CRAO and CRVO could also be the cause of the AF rather than occurring after it. CRAO was strongly associated with carotid artery stenosis and stroke. A Taiwanese population-based study revealed that 19.6% of patients with retinal artery occlusion had a stroke within a 3-year follow-up period, and they had a 2.7-times higher rate of stroke within the first 3 years compared with matched controls [15]. Hollenhorst plaques and venous stasis retinopathy have the highest predictive value for carotid artery stenosis among ocular conditions [6]. The prevalence of retinal emboli at baseline was 1.3% in patients diagnosed as having AF. However, we did not identify any visible retinal artery emboli or venous stasis retinopathy from patient medical records. We discovered that three (1.7%) patients had post-AF NAION, and the condition was not significantly associated with carotid artery stenosis. Similarly, Fry et al. did not report any cases of carotid artery stenosis among patients with AION [16].

Patients with AF usually present to ophthalmologists with isolated visual symptoms. Most study patients (41%) came to the ophthalmologist because of symptoms of AF; this observation was similar to the results of a prior study [17]. Some patients had a concurrent history of hemispheric neurologic symptoms, which might be why these patients did not initially consult ophthalmologists. In our study, some patients (13.3%) had binocular AF symptoms, but laterality was not significantly associated with carotid artery stenosis.

In the study of Biousse and Newman, the duration of visual loss episodes experienced by patients with AF ranged from seconds to 24 h, and only 8% of patients had symptoms lasting longer than 1 h [3]. In our study, 12% of patients had experienced an AF episode that lasted longer than 1 h, and the duration of AF episodes did not differ between patients with or without carotid artery stenosis; this observation is consistent with the findings of Kvickstrom et al. [5]. Some patients had only one episode of transient visual loss, but most (60.1%) of the patients had more than one episode. Similarly, the frequency of AF episodes was not significantly different in AF patients with or without carotid stenosis.

Poole et al. conducted a prospective study of 110 patients with AF to assess mortality and morbidity after AF [10]. Ischemic heart disease was the most common cause of death and occurred at a higher rate than in the general population, and the prevalence of myocardial infarction or failure was 8% after AF [10]. The prevalence of concomitant coronary artery disease in patients with carotid stenosis was as high as 77.1% [18], indicating atherosclerosis is a systemic condition and shares similar factors [19]. Patients with asymptomatic carotid stenosis have about a three-fold higher risk of cardiovascular death or MI compared with a matched population without carotid stenosis [19,20]. In our study, however, MI was only observed in three (1.7%) patients, and all of them were without carotid stenosis. Compared with the aforementioned studies, the prevalence of MI observed was much lower in our study. However, the prevalences of coronary artery disease and ischemic heart disease were significantly higher in patients with carotid stenosis than in those without carotid stenosis (coronary artery disease: stenosis vs. no stenosis = 27.9% vs. 10.7%; ischemic heart disease: stenosis vs. no stenosis = 26.2% vs. 11.6%).

A limitation of our study was that the recorded symptoms of AF were subjectively determined based on patients’ descriptions and a clinician’s interpretation of their symptoms. Some patients had AF symptoms that were not checked by ophthalmologists, and as such, detailed records of ocular examinations were absent. Another limitation was that the diagnosis of carotid artery stenosis was not made based on a single neuroimaging procedure. If TCD or TCCD was not conducted, carotid artery stenosis was diagnosed based on carotid angiography, CTA, or MRA of the brain.

## 6. Conclusions

We observed a high prevalence of carotid stenosis in patients with AF. Meanwhile, concomitant coronary artery disease was also found in patients with carotid stenosis. In particular, carotid stenosis >50% is of great importance to clinicians in detecting a higher risk of stroke and coronary artery disease. In other words, AF attacks are manifestations of internal carotid artery stenosis and are warnings of an impending stroke, and patients with AF should all be checked for carotid stenosis. Older age, male sex, and ischemic heart disease were observed to be independently significant risk factors for carotid stenosis. Regarding ocular diseases, NAION was not a marker for atherosclerotic carotid artery stenosis, whereas CRAO predicted carotid stenosis risk.

## Figures and Tables

**Table 1:** Demographic data on patients with AF (*n* = 173).

	No Carotid Stenosis *n* = 112 (64.7%)	Carotid Stenosis *n* = 61 (35.3%)	*p*-Value
Age (years), Mean ± SD	59.0 ± 16.2	65.9 ± 11.9	0.002
Sex [*n* (%)], 173 (100%)			
Female, 85 (49.1%)	68 (60.7%)	17 (27.9%)	<0.001
Male, 88 (50.9%)	44 (39.3%)	44 (72.1%)	
Smoker [*n* (%)], 36 (20.8%)	16 (14.3%)	20 (32.8%)	0.004
Diabetes mellitus [*n* (%)], 23 (13.3%)	12 (10.7%)	11 (18.0%)	0.176
Hypertension [*n* (%)], 90 (52%)	52 (46.4%)	38 (62.3%)	0.046
Coronary artery disease [*n* (%)], 29 (16.8%)	12 (10.7%)	17 (27.9%)	0.004
Hyperlipidemia [*n* (%)], 53 (30.6%)	29 (25.9%)	24 (39.3%)	0.067
Ischemic heart disease [*n* (%)], 29 (16.8%)	13 (11.6%)	16 (26.2%)	0.014
Prior stroke [*n* (%)], 14 (8.1%)	4 (3.6%)	10 (16.4%)	0.003
Vasculitis [*n* (%)], 0 (0%)	0 (0%)	0 (0%)	
Migraine [*n* (%)], 7 (4%)	7 (6.3%)	0 (0%)	0.046
NAION [*n* (%)], 3 (1.7%)	1 (0.9%)	2 (3.3%)	0.251
Retinal vascular occlusion, 5 (2.9%)	1 (0.9%)	4 (6.6%)	0.012
CRAO, 4 (2.3%)	0 (0%)	4 (6.6%)	
CRVO, 1 (0.6%)	1 (0.9%)	0 (0%)	

Abbreviation: AF: amaurosis fugax, NAION: nonarteritic anterior ischemic optic neuropathy, CRAO: central retinal artery occlusion, CRVO: central retinal vein occlusion.

**Table 2:** Association between symptoms of amaurosis fugax and carotid stenosis.

Symptoms of Amaurosis Fugax	No Carotid Stenosis	Carotid Stenosis	*p*-Value
Laterality, *n* (%)			0.677
One side, 150 (86.7%)	98 (65.3%)	52 (34.7%)	
Both sides, 23 (13.3%)	14 (60.9%)	9 (39.1%)	
Duration, *n* (%)			0.449
≤10 min, 117 (74.5%)	73 (71.6%)	44 (80.0%)	
11~30 min, 21 (13.4%)	16 (15.7%)	5 (9.1%)	
>30 min, 19 (12.1%)	13 (12.7%)	6 (10.9%)	
Frequency, *n* (%)			0.666
1 episode, 104 (60.1%)	66 (63.5%)	38 (36.5%)	
≥2 episodes, 69 (39.9%)	46 (64.7%)	23 (35.3%)	

## Data Availability

The data presented in this study are available on reasonable request from the corresponding author.
